# *QuickStats:* Age-Adjusted Drug Overdose Death Rates* Among Workers Aged 16–64 Years in Usual Occupation^†^ Groups with the Highest Drug Overdose Death Rates — National Vital Statistics System, United States,^§^ 2020

**DOI:** 10.15585/mmwr.mm7129a5

**Published:** 2022-07-22

**Figure:**
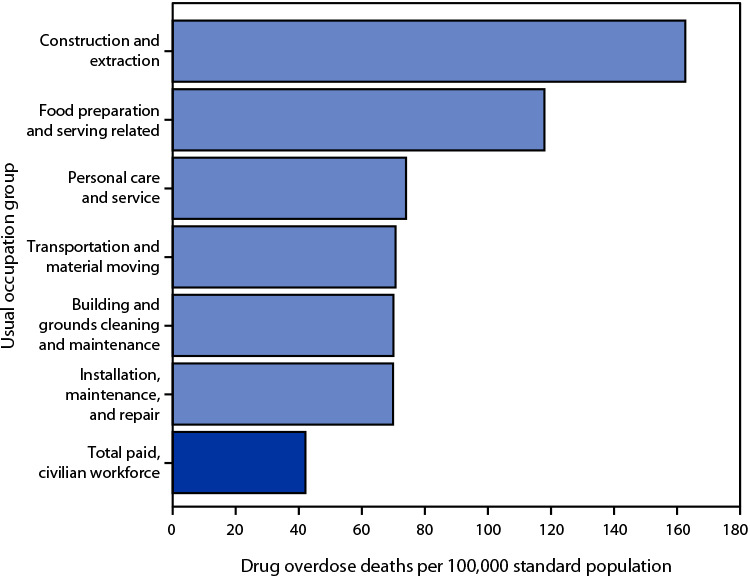
In 2020, the age-adjusted drug overdose death rate among workers with paid, civilian usual occupations was 42.1 deaths per 100,000. Drug overdose death rates were highest among workers in the following occupations: construction and extraction (162.6); food preparation and serving related (117.9); personal care and service (74.0); transportation and material moving (70.7); building and grounds cleaning and maintenance (70.0); and installation, maintenance, and repair (69.9).

**Source:** National Vital Statistics System, Mortality Data. https://www.cdc.gov/nchs/deaths.htm

For more information on this topic, CDC recommends the following links: https://www.cdc.gov/drugoverdose/deaths/index.html and https://www.cdc.gov/niosh/topics/opioids/default.html

